# Early Postoperative Rotational stability and its related factors of a single-piece acrylic toric intraocular lens

**DOI:** 10.1038/s41433-019-0521-0

**Published:** 2019-07-12

**Authors:** Shuyi Li, Xi Li, Suhong He, Qianyin Zheng, Xiang Chen, Xingdi Wu, Wen Xu

**Affiliations:** 1 Eye Center, 2nd Affiliated Hospital of Zhejiang University College of Medicine, Hangzhou, China; 2 Department of Ophthalmology, Affiliated Hangzhou First People’s Hospital, Zhejiang University School of Medicine, Hangzhou, China; 3 Department of Ophthalmology, Shanxi Provincial Cancer Hospital, Affiliated Cancer Hospital of Shanxi Medical University, Taiyuan, China; 4 Suichang Hospital of Traditional Chinese Medicine, Lishui, China; 5 Taizhou Municipal Hospital, Taizhou, China

**Keywords:** Lens diseases, Risk factors, Outcomes research, Lens diseases, Risk factors

## Abstract

**Purpose:**

In the present study, we aimed to evaluate the early postoperative rotational stability of TECNIS toric intraocular lens (IOL) and analyse its correlation with preoperative and intraoperative parameters.

**Methods:**

A total of 102 eyes from 87 cataract patients who underwent implantation of TECNIS toric IOL during July 2016 to November 2017 were enrolled in this retrospective study. Preoperative parameters including corneal astigmatism, axial length (AL), lens thickness (LT), anterior chamber depth (ACD) and sulcus-to-sulcus (STS), were determined. The area of capsulorhexis was measured with Rhinoceros 5.0 software. The follow-up examinations including the residual astigmatism (RAS) and postoperative toric IOL axis, were performed at 1 month and 3 months after surgery.

**Results:**

RAS was −0.84 ± 0.88 D at 1 month and −0.81 ± 0.89 D at 3 months after surgery. The rotation of toric IOL at 3 months was 4.83 ± 3.65°. The Pearson’s r of ACD, horizontal and vertical STS, and toric IOL target axis was 0.011, 0.039, 0.045 and 0.082. The toric IOL rotation was positively correlated with the area of capsulorhexis (*r* = 0.522, *P* = 0.0003), LT (*r* = 0.288, *P* = 0.003) and AL (*r* = 0.259, *P* = 0.009). As for the area of capsulorhexis, the regressive equation was: *y* = 0.682 × −13.105, demonstrating that the diameter of capsulorhexis should be controlled within 5.8 mm to maintain the toric IOL rotation within 5.0°.

**Conclusions:**

TECNIS toric IOLs possessed great early postoperative rotational stability. The area of capsulorhexis, AL and LT were positively correlated with postoperative rotational stability. A capsulorhexis within 5.8 mm had an important significance in improving rotational stability.

## Introduction

As one of the main factors affecting the postoperative visual quality of patients with cataract, astigmatism may lead to visual discomfort, such as blurry vision, visual fatigue and diplopia. In accordance to the survey, patients with cataract have corneal astigmatism to varying degrees. Moreover, 21.3–22.4% of patients with cataract have 1.00 dioptre (D) to 1.50 D of corneal astigmatism, with 10.6% to 12.4% of patients having 1.50 D to 2.00 D and 8.20% to 13.0% of patients having 2.00 D or more [1, 2]. Compared with the conventional approaches, like corneal relaxing incisions (CRI), limbal relaxing incisions (LRI) and laser-assisted in situ keratomileusis (LASIK) [3–5], toric intraocular lens (IOL) has become an increasingly common technique due to its great advantages in predictability, stability and safety.

However, even with precise preoperative calculation and appropriate implantation of toric IOLs, residual astigmatism (RAS) still occurs mainly due to the rotation of toric IOLs. Previous studies have shown that every degree of rotation from the target axis reduces effectiveness by ~3.3%, while >30° of rotation will lead to an increase in astigmatism [6, 7]. Furthermore, material and pattern of IOLs, imbalance of capsular contraction, and viscoelastic residue are associated with the rotational stability of toric IOLs [8–10]. However, the relationship between the early postoperative rotational stability and biometric parameters remains largely unexplored. In our retrospective study, we aimed to evaluate the rotational stability of TECNIS toric IOLs at 3 months after surgery and explore the correlations between biometric parameters and rotational stability.

## Methods

### Study subjects

This retrospective study was performed at the Eye Center, the Second Affiliated Hospital of Zhejiang University. All patients underwent phacoemulsification and implantation with TECNIS toric IOLs during July 2016 to December 2017. TECNIS toric IOLs (AMO, Abbott Park, Illinois, USA) were one-piece, hydrophobic acrylic, aspheric optic IOLs. Patients with cataract who had corneal astigmatism of over 1.0 D on corneal topography (Pentacam, Oculus Optikgeräte GmbH, Wetzlar, Germany) were included in this study. The included patients must have regular corneal astigmatism on Pentacam as the astigmatism meridian of the anterior and posterior surfaces was within 10° and astigmatism power was within 0.75 D. Exclusion criteria included irregular astigmatism, such as forme fruste keratoconus, corneal scars, pterygium, traumatic cataract, glaucoma, retinal detachment, uveitis, macular degeneration or retinopathy, any experience of ocular surgery, or any other comorbidity that might affect postoperative visual function.

### Preoperative assessments

Preoperative assessments were performed, including biometric parameters and toric IOL calculations. Preoperative biometric parameters included corneal astigmatism, axial length (AL), lens thickness (LT), anterior chamber depth (ACD) and sulcus-to-sulcus (STS).

Keratometry was determined using the Pentacam (Oculus Optikgeräte GmbH, Wetzlar, Germany). ACD was measured by anterior segment optical coherence tomography (AS-OCT; Visante OCT, Carl Zeiss Meditec, Inc., Dublin, CA), while horizontal and vertical STS were determined by ultrasound biomicroscopy (UBM, SW-3200 50 MHz UBM Scane, SUOER, China). Additionally, measurements of AL and LT were both performed by A-scan ultrasound. High myopia was defined as AL over 26.00 mm using A-scan ultrasound. Toric IOL power, alignment axis and meridian were calculated using an online calculation software (Abbott Medical Optics, USA; available at https://www.amoeasy.com/calc). A surgically induced astigmatism (SIA) was entered as 0.3 D, and the steep meridian of the cornea was used for the incision position.

### Surgical procedures

Corneal reference marks were made by the same experienced doctor at 3 and 9 o’clock with the subject in an upright, seated position before pupil dilation. All surgeries were performed by the same experienced surgeon (Wen Xu). At the first step of surgery, toric IOL alignment axis was marked using a Mendez ring and highlighted for recognition. A clear corneal incision of 2.0 mm was made, followed by the continuous curvilinear capsulorhexis (CCC), phacoemulsification, irrigation and aspiration of the cortex. The TECNIS toric IOL was inserted and rotated clockwise to approximately 15° short of the intended axis. After viscoelastic material was removed, the IOL was precisely rotated to the target position. Finally, the accuracy of IOL placement was checked, and the incisions were hydrated. The procedures of all surgeries were recorded for further usage of the area of capsulorhexis.

### Postoperative assessments

Patients received examinations at 1 month and 3 months after surgery. The examinations included manifest refraction, keratometry and photographic assessment of toric IOL rotation. After the pupils were totally dilated, the images of toric IOL were captured by a digital camera (Topcon, Tokyo, Japan) connected to the slit-lamp and computer, when two symmetrically marked spots could be clearly recognized on TECNIS toric IOL.

Photographic assessments included the axis of postoperative toric IOL rotation and the area of capsulorhexis during surgery. For each postoperative image, the exact axis of TECNIS toric IOL was calculated using the Adobe Photoshop software. Deviation caused by head tilt, camera rotation or other abnormity was diminished to the most extent with the help of referring to conjunctival vessels. The difference between the exact axis and the target axis demonstrated the early postoperative rotational stability of toric IOL.

**Fig. 1:**
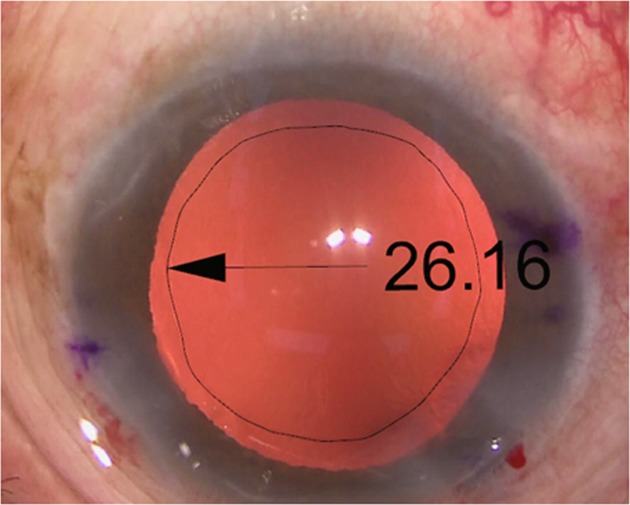
Photograph showing the area of the capsulorhexis after inserting toric IOL calculated by Rhinoceros V.5.0. After importing the image of surgery into Rhinoceros and adjusting to the precise size, the area of capsulorhexis was measured by drawing the line of capsulorhexis. The area of capsulorhexis of this patient was 26.16 mm^2^

The area of capsulorhexis was calculated by using a 3D design software, Rhino V.5.0 (Rhinoceros, Robert McNeel & Assoc, USA). After importing the image of surgery at the step of removing viscoelastic material after implanting toric IOL, the image was resized by adjusting the optic diameter to 6.0 mm. By tracing out the capsulorhexis, the area of capsulorhexis was directly calculated. Such calculation was conducted at least three times, and the mean value was used as the final data (Fig. 1).

### Statistical analysis

Statistical analysis was performed by using SPSS V.11 (SPSS, Chicago, Illinois, USA). Variables are presented as the mean ± standard deviation. Relationships between variables were assessed using Pearson’s correlation analysis. Multiple stepwise regression analysis was applied to assess the independent effects of various factors that might be correlated with toric IOL rotation. *P* values of < 0.05 were considered as statistically significant.

## Results

### Preoperative characteristics

In the present study, a total of 102 eyes from 87 patients, including 51 males and 36 females, were enrolled. The mean age of patients during surgery was 64.38 ± 13.75 years, ranging from 30 to 84 years. The mean AL was 25.17 ± 2.39 mm, while eyes with high myopia accounted for 32.35%. The mean preoperative corneal astigmatism, measured with Pentacam, was 1.80 ± 0.86 D (ranging from 1.00 D to 5.70 D). The mean preoperative toric IOL setting axis was 64.15 ± 52.03°, ranging from 0 to 178°. Table 1 lists all above-mentioned values.

**Table 1:** Preoperative characteristics

Parameters	Value
Age	64.38 ± 13.75 (30–84)
High myopia (%)	32.35 (33/102)
LT (mm)	4.64 ± 0.63 (3.11–6.82)
AL (mm)	25.17 ± 2.39 (21.37–30.23)
ACD (mm)	2.77 ± 0.39 (1.92–3.51)
STS (horizon) (mm)	11.79 ± 0.86 (9.77–13.37)
STS (vertical) (mm)	11.89 ± 0.84 (9.78–13.86)
Corneal curvature (horizon) (D)	43.78 ± 1.58 (40.50–47.00)
Corneal curvature (verticaL) (D)	45.47 ± 1.70 (41.60–48.31)
Corneal astigmatism (D)	1.80 ± 0.86 (1.00–5.70)
Toric IOL degree (D)	−18.54 ± 3.52 (0.00–24.50)
Toric IOL axis (degree)	64.15 ± 52.03 (0–178)

*LT* lens thickness, *AL* axial length, *ACD* anterior chamber depth, *STS* sulcus-to-sulcus, *D* diopters, *IOL* intraocular lens

### Postoperative refractive conditions

The mean RAS was −0.84 ± 0.88 D (ranging from 0 to −2.50 D) at 1 month and −0.81 ± 0.89 D (ranging from 0 to −2.50 D) at 3 months, which was significantly reduced compared with the preoperative corneal astigmatism. No significant differences in RAS were observed between 1 month and 3 months (*P* < 0.01). RAS was 0 D in 27.45% (28/102) of patients, and it was controlled within −0.50 D and −1.00 D in 54.90% (56/102) and 87.25% (89/102) of patients at 3 months, respectively.

### State of toric IOL rotation at 3 months after surgery

The mean toric IOL rotation at 3 months after surgery was 4.83 ± 3.65° (ranging from 0 to 21°). Table 2 shows the distribution of toric IOL rotation at 3 months. Briefly, 68.63% (70/102) of patients rotated within 5° and 95.10% (97/102) of patients rotated within 10°. The maximum of toric IOL rotation was 21°, leading to the greatest RAS of −2.50 D as well.

**Table 2:** Toric IOL rotation 3 months after after toric IOL implantation

Toric IOL rotation (degrees)	N(%)	Clockwise (%)	Counter-clockwise (%)
≤5.0	70 (68.63)	49 (48.04)	14 (13.73)
5.1–10.0	27 (26.47)	16 (15.69)	11 (10.78)
10.1–15.0	3 (2.94)	3 (2.94)	0 (0)
>15.0	2 (1.96)	2 (1.96)	0 (0)

*IOL* intraocular lens

### Factors related with toric IOL rotation at 3 months after surgery

**Fig. 2:**
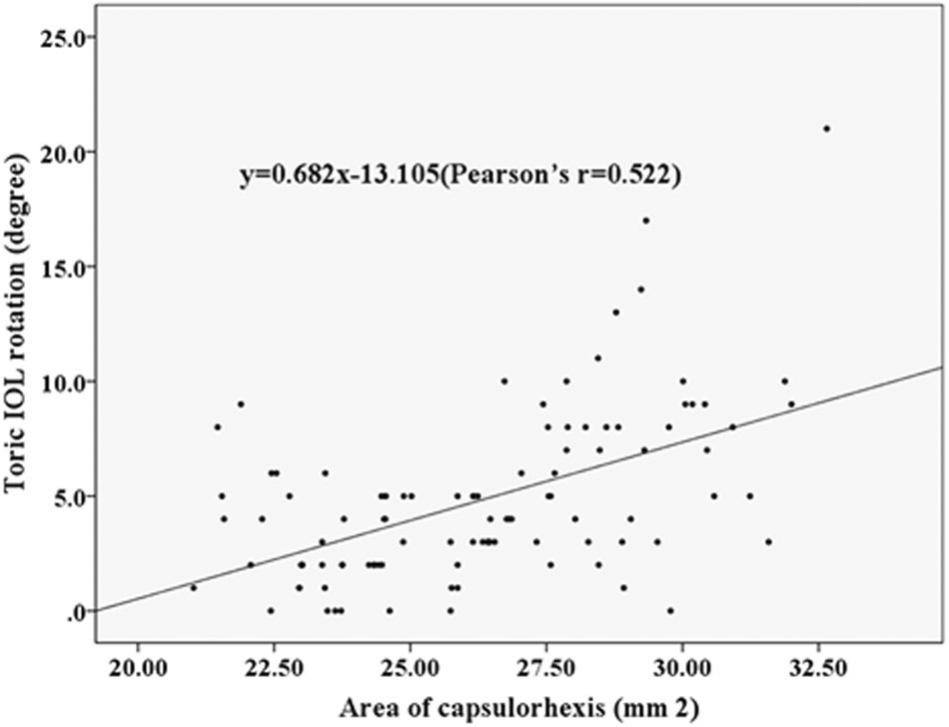
Toric IOL rotation was significantly and positively correlated with the area of capsulorhexis (Pearson’s *r* = 0.522, *P* = 0.0003). *IOL* intraocular lens

The mean area of capsulorhexis was 26.31 ± 2.80 mm^2^ (ranging from 21.02 to 32.65 mm^2^) at 3 months after surgery. Toric IOL rotation was significantly and positively correlated with the area of capsulorhexis. The regression equation was *y* = 0.682 × −13.105 (Fig. 2; Pearson’s *r* = 0.522, *P* = 0.0003). According to the regression equation, when the toric IOL rotation was set at 0°, the area of capsulorhexis was 19.22 mm^2^, corresponding to a CCC of ~4.95 mm in diameter. When the rotation was within 5°, the area of capsulorhexis was 26.54 mm^2^, with a CCC of 5.81 mm. When the area of capsulorhexis was over the limitation, as the diameter of CCC was over 6 mm, the mean toric IOL rotation was significantly increased to 7.81 ± 4.68° (ranging from 0 to 21°), with 66.67% (18/27) of patients over 5°. In conclusion, the diameter of capsulorhexis should be controlled within the range of 5.0–5.80 mm. Simple correlation analysis revealed that AL and LT were positively associated with toric IOL rotation (Table 3, *P* < 0.01). No correlations were found between toric IOL rotation at 3 months and other variables, including ACD, STS (horizon and vertical) and toric IOL axis (Table 3).

**Table 3:** Linear regression analysis of toric IOL rotation as the dependent variables

Variables	Pearson’s *r*	*P* Value
AL	0.259	0.009^a^
LT	0.288	0.003^a^
ACD	0.011	0.940
STS (horizon)	0.039	0.799
STS (vertical)	0.045	0.768
Toric IOL axis	0.082	0.508
Area of capsulorhexis	0.522	0.0003^a^

*LT* lens thickness, *AL* axial length, *ACD* anterior chamber depth, *STS* sulcus-to-sulcus, *D* diopters, *IOL* intraocular lens

^a^Statistically significant correlation

The variables included in the multiple stepwise regression analysis were age, gender, AL, LT, ACD, STS (horizon and vertical), toric IOL axis and area of capsulorhexis. Only area of capsulorhexis, AL and LT were found to be independently correlated with toric IOL rotation at 3-months follow-up (B = 0.321, *P* = 0.022; B = 0.359, *P* = 0.003; B = 1.344, *P* = 0.012, respectively, *R*^2^ = 0.212 and adjusted *R*^2^ = 0.188).

## Discussion

In our present study, the mean RAS was −0.84 D and −0.81 D at 1 month and 3 months, respectively. The RAS of more than half of the patients was controlled within −0.50 D both at 1 month and 3 months. In spite of high proportion of high myopia, our outcome of RAS was still consistent with that of the TECNIS toric IOL model reported by several authors [11–14], probably attributed to our strictly restricted regular corneal astigmatism as the astigmatism meridian of the anterior and posterior surfaces within 10 degrees and astigmatism power within 0.75 D. These results revealed that the TECNIS toric IOLs could successfully maintain refractive cylinder reduction 3 months after implantation into the eyes with preoperative corneal astigmatism.

Effectiveness of toric IOLs has been now highly recognized via hundreds of studies, while the issue of rotational stability still remains unsolved. Therefore, we performed a retrospective review about rotational stability and its potential related factors, including preoperative biometric parameters and surgical techniques. We found that the rotation of TECNIS toric IOL at 3 months after surgery was 4.83 ± 3.65°, ranging from 0 to 21°. In previous studies, the mean absolute misalignment of TECNIS toric IOLs compared with the intended placement at 3 months after surgery ranges from 2.1 to 3.6° at most [11–15], which is slightly better than our results. This might be attributed to the fact that a higher percentage of patients with high myopia (32.35%) have a longer AL. The positive correlation between AL and toric IOL rotation was also observed in our study and in earlier studies [8, 16–18]. Although a higher proportion of patients with high myopia were enrolled, the fact that ~70% of patients were within 5° of rotation could strongly demonstrate the excellent rotational stability of TECNIS toric IOL. Moreover, the direction of rotation was clockwise in the majority of patients, probably due to the design of haptics.

In order to control the situation of axis misalignment, it is highly necessary to explore the potential factors of rotational stability. Apart from the influence of materials and shapes of toric IOLs [19, 20], it is suggested that rotational stability would be correlated with the size of capsular bag. Studies have shown that large-diameter capsular bag tends to be associated with an increase in AL. Moreover, several studies have mentioned the correlation between AL and rotational stability. Most studies have indicated that AL is positively related with axis misalignment [8, 16–18], while Klamann et al. [21] have reported that no significant correlation exists between AL and total absolute rotation. We, for the first time, measured LT using A-scan ultrasound that could directly represent the diameter of capsular bag compared with AL. Our study proved that the correlation between rotation and LT was stronger than that of AL (Pearson’s *r* = 0.288 vs. 0.259). We reviewed AL and LT measured before surgery and found that some patients with high myopia had LT in average as well. This result suggested that it was not advisable to only take AL in consideration. To these patients with high myopia, LT should be calculated before cataract surgery as well. Except for AL and LT, other preoperative biometric parameters, like ACD and STS, did not reveal any significant correlation with rotation of toric IOLs.

An appropriate size of CCC is essential to prevent toric IOL rotation. To the best of our knowledge, our present study was the first one to compare the rotational stability with the accurate size of capsulorhexis. The area of capsulorhexis was significantly associated with rotational stability. When the optic was only partially covered by the anterior capsule, the mean rotation of toric IOLs was evidently increased from 4.83 to 7.81. There was a special case in this study. The toric IOL of this patient was rotated 73° at 1 week and 81° at 1 month after surgery. The IOL repositioning was performed again around 40 days after cataract surgery, after which the IOL remained stable. In this patient, AL was a little longer as 27.07 mm, but the LT was at an average level of 4.50 mm. However, the area of CCC in this case reached 33.06 mm^2^ as the maximum. The optic was completely free of contact between the anterior capsule and IOL in this case. What should be highlighted was that the other eye of this patient was also implanted with a TECNIS toric IOL 1 month prior to the surgery of this eye. In the other eye, with AL of 26.98 mm, LT of 4.47 mm and the area of capsulorhexis of 27.53 mm^2^, IOL misalignment was only 4° at 3 months after surgery. This case highly demonstrated that the size of capsulorhexis played an important role in risk factors of rotational stability.

There are still some limitations in this study. One is the short follow-up time since the underlying capsular shrinking probably occurs at 6 months or 1 year after cataract surgery, though it is reported within the first 3 months in the majority [22]. Another limitation may be the method to calculate the area of capsulorhexis. In several intraoperative photographs, part of capsulorhexis overlapped the edge of toric IOL optic, resulting in deviations when tracing out the capsulorhexis inevitably, even repeated for three times. Moreover, we only calculated the size of capsulorhexis, but did not take IOL contraction into account, which could be measured by the area of overlap between capsulorhexis and optic. Using the VERION digital marker to mark toric IOL alignment axis instead of a Mendez ring can also significantly reduce misorientation of toric IOL [23, 24].

Overall, a single-piece acrylic toric IOL, TECNIS toric IOL, was found to be effective in reducing pre-existing regular corneal astigmatism, and it had good early postoperative rotational stability. Moreover, the rotation of TECNIS toric IOLs was associated with LT, AL and size of capsulorhexis. A capsulorhexis within 5.8 mm had an important significance on improving rotational stability.

### Summary

#### What was known before

- A single-piece acrylic toric IOL, TECNIS toric IOL, showed great early postoperative rotational stability in previous studies.
- Although several studies reported the relationship between rotational stability and axial length, it is still unclear whether preoperative and intraoperative parameters would affect rotational stability.

#### What this study adds

- We found that early postoperative rotational stability of TECNIS toric IOL was correlated with axial length and lens thickness.
- To patients with myopia, lens thickness should be taken into account as well. An appropriate size of capsulorhexis had significantly influence on improving rotational stability of toric IOL as the diameter of 5.8 mm.

## References

[1] Chen W, Zuo C, Chen C (2013). Prevalence of corneal astigmatism before cataract surgery in Chinese patients. J Cataract Refract Surg.

[2] Yuan Xiaoyong, Song Hui, Peng Gang, Hua Xia, Tang Xin (2014). Prevalence of Corneal Astigmatism in Patients before Cataract Surgery in Northern China. Journal of Ophthalmology.

[3] Rigi M, Al-Mohtaseb Z, Weikert MP (2016). Astigmatism correction in cataract surgery: toric intraocular lens placement versus peripheral corneal relaxing incisions. Int Ophthalmol Clin.

[4] Eliwa TF, Abdellatif MK, Hamza II (2016). Effect of limbal relaxing incisions on corneal aberrations. J Refract Surg.

[5] Mohammad-Rabei H, Mohammad-Rabei E, Espandar G (2016). Three methods for correction of astigmatism during phacoemulsification. J Ophthalmic Vis Res.

[6] Mencucci R, Favuzza E, Guerra F (2014). Clinical outcomes and rotational stability of a 4-haptic toric intraocular lens in myopic eyes. J Cataract Refract Surg.

[7] Ma JJ, Tseng SS (2008). Simple method for accurate alignment in toric phakic and aphakic intraocular lens implantation. J Cataract Refract Surg.

[8] Shah GD, Praveen MR, Vasavada AR (2012). Rotational stability of a toric intraocular lens: influence of axial length and alignment in the capsular bag. J Cataract Refract Surg.

[9] Chan CC, Holland EJ (2012). Management of astigmatism: toric intraocular lenses. Int Ophthalmol Clin.

[10] Chassain C, Pagnoulle C, Gobin L (2013). Evaluation of a new intraocular lens platform: centration and rotational stability. Fr Ophthalmo l.

[11] Lee BS, Chang DF (2018). Comparison of the rotational stability of two toric intraocular lenses in 1273 consecutive eyes. Ophthalmology.

[12] Waltz KL, Featherstone K, Tsai L (2015). Clinical outcomes of TECNIS toric intraocular lens implantation after cataract removal in patients with corneal astigmatism. Ophthalmology.

[13] Hirnschall N, Maedel S, Weber M (2014). Rotational stability of a single-piece toric acrylic intraocular lens: a pilot study. Am J Ophthalmol.

[14] Grohlich M, Miháltz K, Lasta M (2017). Evaluation of postoperative astigmatism correction and postoperative rotational stability of two toric intraocular lenses. Klin Monbl Augenheilkd.

[15] Gyöngyössy B, Jirak P, Schönherr U (2017). Long-term rotational stability and visual outcomes of a single-piece hydrophilic acrylic toric IOL: a 1.5-year follow-up. Int J Ophthalmol.

[16] Zhu X, He W, Zhang K (2016). Factors influencing 1-year rotational stability of AcrySof Toric intraocular lenses. Br J Ophthalmol.

[17] Chang DF (2008). Comparative rotational stability of single-piece open-loop acrylic and plate-haptic silicone toric intraocular lenses. J Cataract Refract Surg.

[18] Miyake T, Kamiya K, Amano R (2014). Long-term clinical outcomes of toric intraocular lens implantation in cataract cases with preexisting astigmatism. J Cataract Refract Surg.

[19] Prinz A, Neumayer T, Buehl W (2011). Rotational stability and posterior capsule opacification of a plate-haptic and an open-loop-haptic intraocular lens. J Cataract Refract Surg.

[20] Mukherjee R, Chaudhury K, Das S (2012). Posterior capsular opacification and intraocular lens surface micro-roughness characteristics: an atomic force microscopy study. Micron.

[21] Klamann MK, Von Sonnleithner C, Gonnermann J (2013). Influence of biometric parameters on rotational stability of toric IOLs. Eur J Ophthalmol.

[22] Edrinton TB (2011). A literature review: the impact of rotational stabilization methods on toric soft contact lens performance. Cont Lens Anterior Eye.

[23] Elhofi AH, Helaly HA (2015). Comparison between digital and manual marking for toric intraocular lenses: a randomized trial. Medicine.

[24] Davison JA, Potvin R (2015). Refractive cylinder outcomes after calculating toric intraocular lens cylinder power using total corneal refractive power. Clin Ophthalmol.

